# Non-communicable disease risk factors in patients with major depressive disorders

**DOI:** 10.1192/j.eurpsy.2026.11077

**Published:** 2026-07-31

**Authors:** S. M. Singh, A. Bansal, A. Pal, A. Sharma, V. Suri

**Affiliations:** 1 Psychiatry; 2 Biochemistry; 3 Internal Medicine, PGIMER, Chandigarh, India

## Abstract

**Introduction:**

Introduction: Non-communicable diseases (NCD) are usually chronic and result from a combination of genetic, physiological, environmental and behavioral factors. NCDs are increasingly recognised as significant contributors to morbidity and mortality in developing and developed countries. NCD risk factors can be thought of as variables associated with an increased risk of developing an NCD. NCD risk factor (NCDRF) screening has become prominent owing to the burden of NCDs. Major depressive disorders (MDD) and NCDs seem to share a bidirectional relationship. However data regarding NCDRF is biased towards severe mental illnesses.

**Objectives:**

This study was carried out to assess patients with MDD for NCDRFs.

**Methods:**

Methods: The study protocol was approved by the Institute ethics committee and was carried out in patients attending the outpatient psychiatry clinic of a tertiary hospital in North India. 200 patients (120 males and 80 females) aged 18-69 years with a diagnosis of MDD (not severely depressed at time of assessment) were assessed for socio-demographic and clinical profile and NCDRFs derived from the WHO STEPS framework. Assessment included behavioral profiling, physical measurements and measurements of biochemical parameters. Data were analyzed.

**Results:**

The results indicated that patients with MDD had significant NCDRFs including current and lifetime tobacco and alcohol use, unhealthy dietary patterns, low levels of physical activity, obesity as indicated by body mass index and waist circumference, hypertension and deranged lipid profiles. Results were broadly comparable to epidemiological data in the general population that has been published elsewhere.

**Conclusions:**

NCDRFs may contribute to increased morbidity and mortality seen in MDDs. Assessment of NCDRFs and modification of eligible risk factors should be included in the management of patients with MDD.

**Disclosure of Interest:**

None Declared

